# The Two Pathways: Which Will the World Choose?

**Published:** 1918-01-19

**Authors:** 


### DEDUCTIONS FROM THE WORLD WAR.

### The Two Pathways: Which Will the World Choose?

Among the many existences and occurrences which we fain would know, but which are hidden from us by the thick fog of war, there is probably nothing more vital and of higher interest than the real mind of the German nation. Hints and suggestions and glimpses reach us from time to time through the medium of the daily press, but these are obviously partial and incomplete, and they betray confusion and even mutual contradiction. It may be taken as more than probable that there are sharp divisions both of opinion and ambition, but what are the really prevailing convictions and determinations we really do not know. The doubt refers not only to the present contest, but to the whole outlook on the future scheme of world politics and world government. There are those among us who believe that in the enemy nations there is a mass of opinion which looks anxiously for the inauguration of a reign of universal peace, and for the substitution for armed might of international justice under the sanction of a supreme tribunal.

On the other hand are those who hold that a desire for world domination is still the prevailing attitude of the German people, and that, as the recent past has taught us, to ignore this attitude is dangerously to close our eyes to patent facts. Neither of these views is destitute of support. Each can, indeed, quote facts and declarations in its justification, and, as is the habit of our press guides, quotations are selected as these appear to support the selected conclusion. Thus there exist doubt and uncertainty where it were well we had knowledge. Nor would it appear from recent utterances that those who are fondly believed to possess inner information are much better off than the simple folk who trust for enlightenment to the daily press. Even President Wilson is driven to ask whether the official spokesmen really express the actual meaning of the nation they profess to represent. The doubt thus has much more importance than a mere cloud on curiosity. It becomes an obstacle to understanding and to the possibility of adjustments. From all this it would seem a fair inference that both the pictures of German opinion which are painted for us have a substantial element of truth—that, in short, the nation is divided into two groups which struggle with one another for mastery. Not that this leads us much farther, for we do not know the relative strengths of these groups, nor the prospect which one or the other has of making its views prevail. But if we accept this conclusion we do get an explanation of one of the prominent facts of the hour—namely, the reluctance of the German politicians to state with precision and clearness the war aims which they cultivate. On our own side, whatever may be said of the past, there is no doubt now of the ends we intend; and there is no doubt that behind there is the resolution of the whole nation. We have lately had from the most authoritative source a definition of resolves which had secured in advance the approval of all classes and all parties; and the programme has been endorsed in substance by the nations allied with us. The enterprise contemplates much more than victory in the present contest. It looks to a new world order, and to a plan in which humanity at large is vitally concerned. The ambition is not a mere adjustment of the present quarrel, but a scheme of moral discipline which shall regulate and control the lives of the nations for all time to come. What is in debate at the present moment is the fate of the world, for between the ideal of abiding peace and an aggravation of the ills involved in a universal preparation for war there is no half-way house. Either we must secure our aims, or ourselves and other nations must return to strenuous organisation for the next war, and to the certainty that the present horrors will repeat themselves in accentuated measure. The human race has to choose which of these two masters it will serve, and the choice is indeed a fateful one. By the free nations this choice has already been taken. But the will depends on opportunity. And the opportunity is conditioned by the mental attitude of the German nation. If the will to power carries it on the will to justice there is an end of the discussion. Here then is the motive which makes us desire earnestly an authoritative voice which would declare truthfully and completely the real relation of our enemies to the world problems of the future. As a united people we have spoken our word. So far as we can judge across the din of war there is as yet no equally agreed and unambiguous reply.

It happens, however, that we are not quite without light on the issue which so greatly concerns the entire human race. The latest, and by no means the least significant, of the gleams of this order has just appeared in the translation of a book* written by one of Germany's most distinguished soldiers. The writer occupies one of the highest ranks in the military hierarchy, and his professed purpose is to gather from the experiences of the present contest lessons which will guide his country to greater efficiency for war in the future. That war can be abolished is a proposition which he thinks hardly worthy of discussion, and he declares that an ambition to this end is an idle and sentimental dream. "War has its basis in human nature," he writes, "and as long as human nature remains unaltered, war will continue to exist." Nor will he allow that war is an unmixed evil. On the contrary, his verdict is that "the effects of war are in many respects very beneficial. War banishes pretence and reveals the truth." As for a league of nations to maintain and enforce the international control of disputes he will have none of it. "We misconstrue reality," he says, "if we imagine it is possible to rid the world of war by means of mutual

* *Deductions from the World War.* By Lieutenant-General Baron von Freytag-Loringhoven. London: Constable and Co., Ltd. Pp. 178 + vi. Price 2s. 6d. net.

**WHICH PATHWAY WILL THE WORLD CHOOSE?**—(*continued*).

agreements." These he proclaims as merely "treaties which will not on every occasion be capable of holding in check the forces seething within the States." "Therefore," he adds, "the idea of a universal league for the preservation of peace remains a Utopia, and would be felt as an intolerable tutelage by any great and proud-spirited nation." There is here, it will be observed, no lack of plain speech and no desire to conceal the fashion of policy advocated. The argument is that war preparation for the future is a national necessity, and that certain experiences of the war of to-day show that this preparation must be more intensive and more complete, if, on a later occasion, Germany is to obtain the world power which is due to her; and this argument accepts and rests on the premises which are defined in the sentences just quoted. No achievement in other fields will avail. It is with the test of war that "our world position stands or falls," and "Germany must for all time to come maintain her claim to world power." These statements have the virtue of frankness, though apparently they were intended only for the writer's fellow-countrymen.

The significance of these views is heightened by the position which their author occupies, and it may fairly be said that further emphasis is lent to them by the whole tone and manner of the book in which they appear. Here is no wild, irresponsible, screaming and mind-blinded patriotism. On the contrary, the argument is conducted with entire reasonableness and is advanced by one who has looked with a thoughtful mind at all the issues raised by the war and at the problems which the whole of the international position presents. With rare exceptions he recognises the achievements of his opponents, and is at no pains to conceal the blunders and disappointments which the war has brought to his own countrymen. His most serious lapse from this position is when he represents the United States as influenced purely by "business pacificism" and by "crass materialism," and here he plainly affords a glaring illustration of the inability of even the most intellectual Germans to understand the psychology of other nations. Whatever else may be true about the war, it is at least certain, that, the Americans have come into it under the sway of a passionate ideal and that the words of their President aptly represent the national conviction. He pays tribute to the "lofty national purpose" of the French and to the "tough courage" and the "efficiency" of the English, but some of his remarks on his enemies will certainly excite surprise. "Deeds of horror and senseless rage of destruction," "brutality and distressing excesses in the treatment of prisoners" have indeed marked the course of the war, but it is news to hear that such things have distinguished the conduct of the Allies. We find the real criminals in quite another quarter, and if we agree with our author that "the vast difference between civilisation and *Kultur* has been clearly revealed" we find a meaning in the sentence exactly opposed to the one which he intends. But subject to these are a few similar qualifications the book is marked by a spirit of detachment and calm reason. Its author is evidently a man of capacity, a thorough student of his profession and of the relation of war to world affairs. Accept his standpoint and the conclusions which he advances can hardly be gainsaid. Nor does he shrink from the full consequences of his claim. More money for more soldiers and more warlike appliances must be found by the State; frontiers must be armed by special means of defence; Germany for all time to come must maintain her claim to sea-power; the training of youth must be related to the development of German armed force; even in peace there must be kept vividly before the national mind "the grandeur and sublimity of the soldier's calling"; and war to a growing extent must be directed against the whole of the national life of the enemy. Such are some of the outcomes which are here candidly displayed as the inevitable teachings of the present war, assuming, as the writer does, that in the future, as in the past, every nation, which intends to strive for the expression of power in the world, must be ready to enforce its will through the supreme test of battle. The story is a plain one, and gratitude is due to the publishers for having given it to the English reader. It cannot be said that we have not had fair warning if, as seems probable, the military party in Germany manages to retain its predominance.

Of course it may be said that the teaching and doctrines here to be found are those of a professional soldier, whose natural disposition is to believe in the value and capacities of his profession. The writer, however, is evidently something more than a fighting man. He looks out on the world and human nature and he sees himself compelled, not without reluctance, to accept the views which he here advocates. Were a "spiritual transformation of the human race" within sight he might be ready to "dispense with might." But he sees no such prospect above the horizon, and on the basis of human history he builds his present conviction. We may take a lesson from him here. Books written by soldiers, and readily to be compared with the present one, circulated widely in Germany before the war, and no one questions their educational influence on the German people. Will not this experience be repeated? The answer cannot be doubted. Will the educational value be appreciated also by the British peoples? The picture is plain enough, and we owe the writer thanks for his frankness. Over and against his scheme for the future is the one which has been presented by the leaders of the Allies. The two obviously cannot live in one and the same world. It is much to have the contrast set in such sharp terms. Either one or the other may be chosen, but not an alternation or combination of the two. War must either be abolished or must become the abiding preoccupation of mankind. This is the issue, and Freytag-Loringhoven's book ought to dissipate any doubts on the matter. For this he deserves our thanks, and we believe the more widely circulated is his book the more resolved the world will be to bring his doctrines to the dust.

